# Characteristics of Ship-Emitted VOCs and Their Contributions to Urban Atmospheric VOCs in Guangzhou, China

**DOI:** 10.3390/toxics13060479

**Published:** 2025-06-05

**Authors:** Xueying Zeng, Liwei Wang, Haining Wu, Chenglei Pei, Hong Ju, Junjie He, Ming Liu, Mei Li, Daiwei Chen, Yongjiang Xu, Wenlong Tang, Jinchi Li, Chunlei Cheng

**Affiliations:** 1 College of Environment and Climate, Institute of Mass Spectrometry and Atmospheric Environment, Guangdong Provincial Engineering Research Center for Online Source Apportionment System of Air Pollution, Jinan University, Guangzhou 510632, China; zengxueying2022@163.com (X.Z.); chendv2025@163.com (D.C.); xuyongjiang2022@163.com (Y.X.); tangwenlong0108@163.com (W.T.); ljc13100209679@163.com (J.L.); 2 Guangdong-Hongkong-Macau Joint Laboratory of Collaborative Innovation for Environmental Quality, Guangzhou 510632, China; 3 Jiaxing Ecological Environment Emergency Monitoring and Accident Investigation Center, Jiaxing 314000, China; 13575300099@163.com; 4 Guangzhou Maritime Safety Administration, Guangzhou 510700, China; wuhaining_gz@163.com; 5 Guangzhou Ecological and Environmental Monitoring Center of Guangdong Province, Guangzhou 510030, China; peichenglei@163.com; 6 Guangzhou Sub-Branch of Guangdong Ecological and Environmental Monitoring Center, Guangzhou 510006, China; juh@gz.gov.cn (H.J.); hjj2225833@126.com (J.H.); 7 Guangzhou Hexin Instrument Co., Ltd., Guangzhou 510530, China; lm3.1415@hotmail.com

**Keywords:** VOCs, ship emissions, source apportionment, port atmospheric research, ozone

## Abstract

With the implementation of China’s low-sulfur fuel policy, the characteristics of volatile organic compounds (VOCs) emitted from ship exhausts have changed significantly, and the influence of these emissions on the local atmosphere of port cities needs to be evaluated. In this study, the characteristics of localized source profiles of ship-emitted VOCs with respect to different ship types, fuel types, and engine operating conditions were analyzed in Guangzhou Port. Oxygenated VOCs (OVOCs) dominated in ferry (91.1%), cargo ship (87.0%), and tugboat (54.4% ± 7.9%) emissions (diesel fuel), while alkanes (56.3% ± 1.6%) and alkenes (36.0% ± 0.9%) were major species in multi-purpose ship (LNG fuel) emissions. These results suggest the dominance of OVOCs in the exhaust emissions of diesel-type ships and the prominent difference in ship-emitted VOCs between diesel and LNG fuel ships, which also influenced the emission characteristics of VOCs from main and auxiliary engines. Based on the measured source profiles, ship emissions contributed 18.2% ± 0.8% (summer), 8.7% ± 1.9% (winter), 6.0% ± 1.1% (spring), and 5.6% ± 1.7% (autumn) to VOCs in the port area, and 7.8% ± 1.5% in July and 5.0% ± 0.5% in September in the urban area. An air mass trajectory analysis revealed that the south wind transported the ship exhaust emissions to the port area and inland urban area, which explained the higher contributions of ship emissions in summer and more ship emissions received in the port area than in the urban area. Therefore, estimating the influence of ship emissions on ambient air quality in port cities requires integrating local ship source profiles, locations, and meteorological conditions. This study provides insights into the ship-emitted VOC characteristics given China’s low-sulfur fuel policy and their differential contributions to urban atmospheric VOCs.

## 1. Introduction

According to statistics from the United Nations Conference on Trade and Development (2023), shipping accounts for over 80% of global trade and 60% of international trade [1]. Moreover, maritime trade is expected to increase about three times from 2019 to 2050 (https://www.oecd.org/en/topics/ocean.html (accessed on 15 January 2025)). By the end of 2020, Chinese international shipping trade accounted for one-third of global shipping trade, and the capacity of its maritime shipping fleet reached 314 million tons [2]. Meanwhile, the navigable mileage of inland waterways in China had reached 127,700 km by the end of 2020, ranking first in the world. In 2023, cargo throughput at Guangzhou Port reached 675 million tons, ranking fifth in the world [3]. Ship emissions are important sources of atmospheric particulate matter (PM_10_ and PM_2.5_) and gaseous pollutants (SO_2_, NOx, VOCs, and CO) [4,5]. In 2018, ship exhausts emitted 9.6–10.9 million tons of SO_2_, 16.7–20 million tons of NOx, 1.4–1.9 million tons of PM_2.5_, and approximately 5.3 million tons of non-methane volatile organic compounds (NMVOCs) into the atmosphere, accounting for 9.2%, 16.8%, 4%, and 4% of the total global anthropogenic emissions, respectively [6]. Ship-emitted NOx, VOCs, and CO can influence the concentrations of tropospheric O_3_, which impacts the regional air quality and atmospheric oxidation capacity [7,8]. In recent years, the International Maritime Organization (IMO) has formulated regulations to limit SO_2_ and NOx emissions within emission control areas (ECAs) [9]. In 2015, China established domestic emission control areas (DECAs) within a 12-nautical-mile range along the coasts of the Bohai Rim, the Yangtze River Delta, and the Pearl River Delta (PRD) regions. Since 1 January 2019, international voyaging ships must use fuel with sulfur content of no more than 0.5% m/m when entering DECAs. Furthermore, international voyaging ships must use fuel with a sulfur content of no more than 0.1% m/m when entering the inland waterway control area after 1 January 2020 [10].

Previous studies have found that, after the low-sulfur fuel policy was implemented, SO_2_ and PM emissions from ship exhausts significantly decreased; however, VOC emissions are still increasing [11,12]. Moreover, as the VOCs emitted by ships contain a higher proportion of high-carbon alkanes (C > 9) and aromatic hydrocarbons (such as benzene, toluene, and xylene), their ozone formation potential (OFP) and secondary organic aerosol formation potential (SOAFP) are generally higher than those of VOCs emitted by road vehicles [13,14]. Given the strict control of land-based VOC emissions, increasing VOC emissions of ship exhausts may become increasingly significant for O_3_ and PM_2.5_ formation in port areas [15]. At present, heavy fuel oil (HFO) and marine diesel oil (MDO) are the most commonly used fuels for ships in China; notably, VOC components can vary between different fuel types. For example, Liu et al. (2022) found that since the distillate of marine gas oil (MGO) is lower than that of HFO, the alkanes emitted by MGO are more volatile [16]. In addition, studies mainly focused on the characteristics of NMHCs emitted by ships [17,18,19,20,21]; VOC emission factors (EFs) and emission source profiles under different engine operating conditions and from different ship types have been scarcely reported [2,22,23,24]. Indeed, the low-sulfur fuel policy has changed the VOC emission characteristics of these different ships.

Field measurements were conducted on the characteristics of ship-emitted VOCs across different regions and vessel types before the implementation of the low-sulfur fuel policy. Zhu et al. (2018) investigated the VOC compositions of diesel fuel ship emissions in the Yangtze River Delta, revealing alkenes and alkanes as the dominant components, including ethylene, ethane, acetylene, and propane [25]. In Zhoushan Port, Wang et al. (2018) constructed localized VOC source profiles of ship emissions and off-road mobile sources, identifying alkanes as their primary components [26]. Another study in the PRD by Wang et al. (2020) demonstrated that oxygenated VOCs (OVOCs) constituted 48.9–67.3% of diesel fuel ship emissions, with formaldehyde and acetaldehyde being predominant in cargo ships and speedboats, respectively [24]. The low-sulfur fuel policy has significantly changed VOC emission profiles from ship exhaust. Xiao et al. (2018) investigated VOCs emitted from container ships at Jingtang Port, finding alkanes and aromatics were dominant in VOCs, particularly benzene, toluene, and C_7_-C_9_ alkanes [13]. Wu et al. (2020) conducted field studies of cargo- and container-ship-emitted VOCs in the PRD and revealed a 15-fold increase in VOC emission factors since the low-sulfur fuel policy was implemented [11]. They also found that reactive alkenes (including ethylene, propylene, and isobutane) had replaced alkanes as the dominant components. Zhang et al. (2024) measured VOCs emitted from low-sulfur fuel vessels [14]. They found aromatic VOC profiles changed with the ship type, showing that ocean-going ships exhibited elevated naphthalene levels compared with coastal vessels’ benzene–toluene–xylene dominance. These studies indicate that the characteristics of VOCs emitted by ships in different ports exhibit significant differences with ship type, tonnage, and fuel type. Although many studies have focused on the VOC emission characteristics of large container ships and ocean-going vessels, VOCs from small cargo ships in ports and inland waterways pose more of a threat to urban air quality. Additionally, compared with diesel-powered ships, liquefied natural gas (LNG)-fueled ships have greater advantages in terms of clean emissions of atmospheric particulates, along with NOx and SO_2_, leading to the increasing use of these ships. In summary, the composition of VOCs emitted by ships is influenced by multiple factors; thus, authentic and accurate local VOC source profiles from ship emissions are essential to accurately assess the contribution of ship-emitted VOCs to the atmosphere in port cities. At present, there are no detailed source profiles of ship-emitted VOCs in Guangzhou, and their contribution to the ambient atmospheric VOCs in the Guangzhou Port Area is unclear.

In this study, VOCs emitted from four types of ships (ferries, cargo ships, tugboats, and multi-purpose ships) were assessed in Guangzhou Port. The characteristics of the localized source profiles of ship-emitted VOCs with respect to different ship types, different fuel types, and different engine operating conditions were investigated. In addition, real-time observations of ambient VOCs were conducted at the port and urban areas. Based on the measured characteristics of source-emitted VOCs, the contributions of ship emissions to the ambient VOCs in the port and urban areas were determined and comparatively studied. These results provide insights into the influence of ship-emitted VOCs on the atmospheric loadings of VOCs in port cities and will help formulate effective control strategies.

## 2. Sampling and Data Analysis

### 2.1. Samples Collection

**Figure 1:**
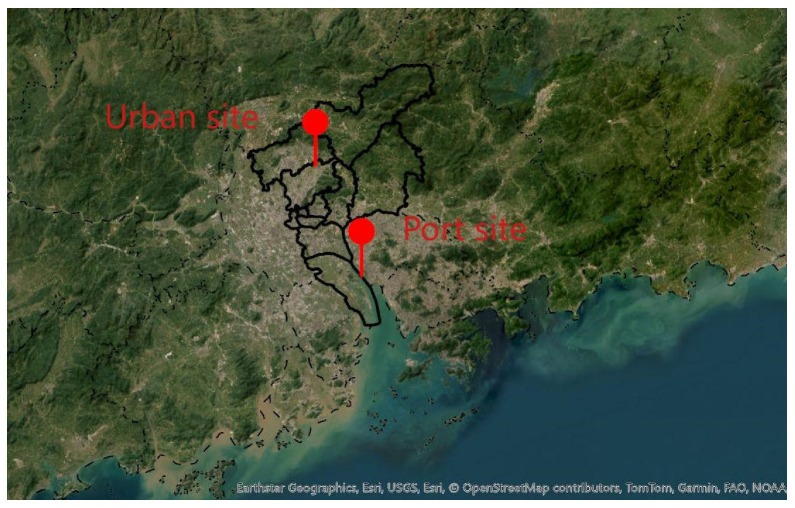
The location of the sampling sites for ambient atmospheric VOCs in the port site and urban site in Guangzhou.

In this study, source-emitted VOCs were sampled from five ships (including one ferry, one cargo ship, two tugboats, and one multi-purpose ship) at Huangpu Port in Guangzhou (Figure 1); the information on each ship is shown in Table 1. VOC samples were collected using stainless steel canisters (Hangzhou Tianjing Detection Technology Co., Ltd., Hangzhou, China, model HHJ6000, capacity 6 L). Before sampling, all canisters were cleaned with nitrogen (N_2_, 99.999%) at least five times and then evacuated under −30 psi to ensure there were no residual pollutants inside the canisters. After sampling, the canister pressure was checked to ensure it returned to 0 psi. The OVOC components from the ship exhaust were collected using 2,4-Dinitrophenylhydrazine (DNPH) columns (Waters Corporation, MA, USA, model WAT037500). All sampling was conducted during the actual operation of the ships, and samples were collected when the ship’s main engine and auxiliary engine were in operation. The exhaust was sampled by inserting a stainless steel tube into the exhaust port. The insertion depth was about 5 cm, ensuring that the sampling tube did not touch the inner wall of the chimney. Then, the sampling tube was connected to a dilution channel through a silicone tube. After dilution, the exhaust gas entered the canister through a flow-limiting valve to collect VOCs (flow rate: 12 L/h; sampling time: approximately 30 min). Meanwhile, the exhaust gas passed through the silicone tube and an ozone-removal column and then entered the DNPH column, which was connected to a pump (flow rate: 0.5 L/min; sampling time: 15 min) to complete OVOC collection. Before sampling, the DNPH columns were stored in a refrigerator at 4 °C away from light, and they were wrapped with aluminum foil during the sampling process. After sampling, they were sealed and refrigerated at below 4 °C and analyzed within 30 days. During sampling, one set sample (including one canister, one DNPH column, and one blank) was separately collected from the main engine and auxiliary engine. Due to the limitation set by the onboard operation time of the in situ measurement and the available ship types, one set sample each was obtained from a ferry ship and a cargo ship, and two set samples were collected from two tugboats and one multi-purpose ship. Nevertheless, the ferry, cargo ship, and tugboats all use diesel fuel, and the averaged characteristics of their VOC emissions still represent most diesel-type ship emissions. These can be used to resolve their contributions to ambient atmospheric VOC concentrations.

Online observations of ambient atmospheric VOCs were conducted at two sites (Figure 1): the port site (113°60′ E, 22°77′ N) and the urban site (113°35′ E, 23°37′ N). The online VOC measurements were carried out at the port site from September 2022 to August 2023, while the measurements at the urban site were conducted for two months between July and September 2022. In all, 8760 sets of VOC data were obtained at the port site, and 1464 sets of VOC data were collected at the urban site. The VOCs were determined using AC-GCMS (model 1000, Guangzhou Hexin Instrument Co., Ltd., Guangzhou, China).

### 2.2. Analysis of VOCs and OVOCs

The VOCs and OVOCs were analyzed according to methods from the United States Environmental Protection Agency. The offline VOC samples were also measured using AC-GCMS, which was equipped with dual injection ports, dual chromatographic columns, and dual detectors (FID and MS). GC-FID was used to detect low-carbon compounds, from C_2_ to C_4_, while MS was used to detect high-carbon VOC components, from C_5_ to C_12_. A total of 117 types of VOCs were detected, including 13 kinds of aldehyde and ketone compounds [27,28]. The OVOC samples collected using the DNPH column were detected with a high-performance liquid chromatography system (LC-MS, model 2000, Guangzhou Hexin Instrument Co., Ltd.), which measured 11 compounds, namely formaldehyde, glyoxal, cyclohexanone, isovaleraldehyde, *p*-toluic aldehyde, methylglyoxal, 2,5-dimethylbenzaldehyde, heptanal, octanal, nonanal, and decanal. The total OVOCs reported in this study included 24 species measured using both AC-GCMS and HPLC.

### 2.3. QA/QC

Quality assurance and quality control (QA/QC) procedures were strictly followed throughout the collection, transportation, and storage of VOC samples through the proper use of canisters, ozone-removal columns, and DNPH columns. Prior to sampling, all canisters were cleaned at least five times with ultrapure nitrogen (N_2_, 99.999%) and subsequently evacuated to −30 psi to ensure the removal of any contaminants. The pressure in each canister was maintained at −30 psi before sampling and rechecked after sampling to confirm it had returned to 0 psi. Standard curves for VOC analysis were constructed using six concentration gradients: 0.8, 1.6, 2.4, 4.0, 6.4, and 8.0 ppb. During calibration, the correlation coefficient (r) had to be higher than 0.99, or the relative standard deviation (RSD) of the six gradient concentrations for each VOC compound had to be less than 30%; otherwise, the calibration curve needed to be reconstructed. An OVOC standard curve with r ≥ 0.99 was constructed using six concentration gradients: 0.1, 0.2, 0.4, 0.6, 0.8, and 1.0 mg/L. Quality control requirements for the VOCs included the following: the standard deviation between the standard concentration and the test concentration had to be less than 10%, and the standard deviation between the standard peak retention time and the test peak retention time had to be less than 5%. These requirements ensured the accuracy and reproducibility of VOC measurement results.

### 2.4. Data Analysis

#### 2.4.1. VOC Source Profile Construction

This study measured component fingerprints of VOC emissions from different types of ships. By quantitatively examining the concentration distribution of individual VOC components and the abundance profiles of each species, VOC source profiles of each ship type were constructed using a normalization approach. The methodology for building VOC source profiles was as follows: First, calculate the mass percentage of individual VOC components relative to the total VOC mass concentration. Then, normalize the mass concentration of each sample (relative to the sample’s total VOC mass concentration). Finally, average the mass percentages of identical VOC components across the same type of emission source.

$$f_{ik}=\frac{C_{ik}}{C_{sum,k}}$$

where *f_ik_* is the mass percentage of the *i* species in sample *k* (%); *C_ik_* is the mass concentration of the *i* species in sample *k* (ppb); and *C_sum,k_* is the total mass concentration of sample *k* (ppb).

$$f_{i}=\frac{\sum_{k=1}^{n}f_{ik}}{n}$$

where *f_i_* is the mass percentage of the *i* species (%), and *n* denotes the number of samples.

#### 2.4.2. Calculation of Ozone Formation Potential

Ozone formation potential (OFP) is used to quantify the maximum contribution of different VOCs to ozone generation under optimal reaction conditions. Calculating OFP is based on the relationship between the concentration of the target VOCs and their maximum incremental reactivity (MIR), as expressed in the following formula:

$$OFP_{i}=MIR_{i}\times C_{i}$$

where $OFP_{i}$ is the contribution of the target VOC *i* to ozone production (μg/m^3^); *C_i_* is the mass concentration of the VOC (μg/m^3^); and *MIR_i_* of the compound, defined as the mass of ozone produced per unit mass of VOC consumed in photochemical reactions (μg O_3_/μg VOC). *MIR_i_* values are experimentally determined and widely used in atmospheric photochemical process assessments [29,30].

#### 2.4.3. Potential Source Contribution Function Analysis

The potential source contribution function (PSCF) is a regional pollution source identification method based on the hybrid single-particle Lagrangian integrated trajectory model. By analyzing air mass trajectory data, it can assess the contributions of pollutants from different regions to a target site. This method is widely applied in the discussion of atmospheric pollution source tracing and transport.

In this study, 72 h backward trajectories at an altitude of 1000 m above the sampling site were calculated, aiming to trace the potential sources of air masses. The ij-th component of the PSCF field is expressed as

PSCFij=mijnij

where *m_ij_* is the total number of trajectory endpoints within the same grid cell where the measured compound concentration exceeds the predetermined threshold for that compound. *n_ij_* is the total number of all trajectory endpoints passing through the grid cell (*i*, *j*) (divided into 0.5° × 0.5° latitude and longitude grid cells). Since the bias in the *PSCF* results increases with the distance of the grid cell from the receptor site, to mitigate the impact of uncertainty in grid cells with small *m_ij_* values, the *PSCF* value is multiplied by a weighting factor *W_ij_* (hereinafter referred to as WPSCF) to obtain the weighted potential source contribution function. *W_ij_* is an empirically determined weighting value based on the number of air mass trajectory endpoints in the grid cell [31]. The average of the numbers of trajectories that passed through all cells is expressed as *N_ave_*. *W_ij_* is defined as follows:

$$W_{ij}(year)=\left\{\begin{array}{c} \begin{array}{cc} 1.00 & N_{ij}\geq 80 \end{array}\\ \begin{array}{cc} 0.70 & 80>N_{ij}>40 \end{array}\\ \begin{array}{c} \begin{array}{cc} 0.40 & 40>N_{ij}>20 \end{array}\\ \begin{array}{cc} 0.20 & 20>N_{ij} \end{array} \end{array} \end{array}\right.$$

$$W_{ij}(season)=\left\{\begin{array}{c} \begin{array}{cc} 1.00 & N_{ij}\geq 3N_{ave} \end{array}\\ \begin{array}{cc} 0.70 & 3N_{ave}>N_{ij}>\frac{3}{2}N_{ave} \end{array}\\ \begin{array}{c} \begin{array}{cc} 0.40 & \frac{3}{2}N_{ave}>N_{ij}>N_{ave} \end{array}\\ \begin{array}{cc} 0.20 & N_{ave}>N_{ij} \end{array} \end{array} \end{array}\right.$$

#### 2.4.4. PMF Analysis

In this study, positive matrix factorization (PMF) was used to estimate the contributions of different sources to VOCs [32]. First, the uncertainties of individual chemical components were determined using weighting, and, then, constrained least squares fitting was applied to identify the major pollution sources and their contribution percentages:

$$x_{ij}=\overset{p}{\underset{k=1}{\sum }}g_{ik}f_{kj}+e_{ij}$$

where *x_ij_* represents the concentration of the *j*-th species in the *i*-th chemical sample; *g_ik_* represents the contribution of the *k*-th source to the *i*-th chemical sample; *f_kj_* represents the score matrix of the *j*-th species on the *k*-th source factor (or the source profile matrix element); *e_ij_* represents the residual for the *j*-th species in the *i*-th chemical sample; and *p* represents the total number of independent sources. PMF ensures the source contribution, *g_ik_*, for each sample is non-negative. It then minimizes the objective function, *Q*, which is based on the residuals, *e_ij_*, and the uncertainties, *u_ij_*, of the samples. The optimal solution in PMF is achieved by minimizing the objective function, *Q*, solving for the factor matrices, *G* (containing *g_ik_*) and *F* (containing *f_kj_*), which yield a small *Q* value.

$$Q=\overset{n}{\underset{i=1}{\sum }}\overset{m}{\underset{j=1}{\sum }}{\left[\frac{e_{ij}}{u_{ij}}\right]}^{2}=\overset{n}{\underset{i=1}{\sum }}\overset{m}{\underset{j=1}{\sum }}{\left[\frac{x_{if}-\sum_{k=1}^{p}g_{ik}f_{kj}}{u_{ij}}\right]}^{2}$$

*uij* represents the uncertainty in concentration. *Q*(*true*) includes all points, while *Q*(*robust*) excludes points poorly fit by the model. Since *Q*(*robust*) is unaffected by points where the PMF fit is poor, it is used as the key parameter to select the best run from multiple runs. In this study, missing values and zero values are replaced by the median of the corresponding species, with the corresponding uncertainty set to four times the median. If a species’ concentration is below the method detection limit (*MDL*), the concentration is replaced by one-half of the *MDL*, with the corresponding uncertainty set to five-sixths of the median. For species with concentrations above the *MDL*, the species concentration is the observed value, and its uncertainty is calculated as follows:

$$Unc=\sqrt{({\left(EF*c\right)}^{2}+{\left(\frac{1}{2}MDL\right)}^{2}})$$

where *c* represents the species concentration, *Unc* represents the uncertainty, *EF* represents the error fraction (typically the instrument’s RSD value), and *MDL* represents the method detection limit.

## 3. Results and Discussion

### 3.1. The Source Profile of Ship-Emitted VOCs

**Figure 2:**
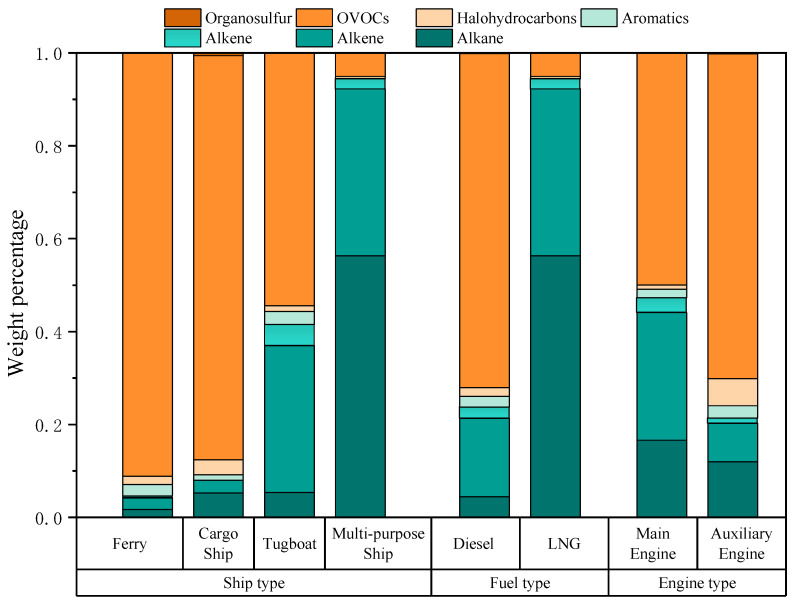
The characteristics of VOCs in ship exhaust from different types of ships, fuels, and engine sources. The concentration of each component is presented as its percentage in the total measured VOCs.

In this study, VOC source samples from four types of ships were collected to construct ship source profiles. The measured VOC species contained seven categories: alkanes, alkenes, alkynes, aromatic hydrocarbons, halogenated hydrocarbons, OVOCs, and organic sulfur compounds. The VOC components and mass fractions emitted by these four ship types are shown in Figure 2. For the ferry, the top three VOCs were OVOCs (91.1%), alkenes (2.5%), and aromatic hydrocarbons (2.5%). For the cargo ship, the top three were OVOCs (87.0%), alkanes (5.3%), and halogenated hydrocarbons (3.2%). For the tugboats, the top three were OVOCs (54.4% ± 7.9%), alkenes (31.6% ± 7.0%), and alkanes (5.4% ± 1.1%). For the multi-purpose ship, the top three were alkanes (56.3% ± 1.6%), alkenes (36.0% ± 0.9%), and OVOCs (5.0% ± 0.1%). Overall, due to differences in fuels used by the four ship types, the proportion of OVOCs in the VOCs emitted by the diesel-fueled ships was higher than 50%, while the exhaust of the LNG-fueled ship was dominated by alkanes. Reported studies on VOCs emitted by cargo ships in the PRD also showed that OVOCs had the highest proportion (48.9–67.3% of total VOCs) [24], which was consistent with the measurement results for cargo ship emissions in this study.

**Figure 3:**
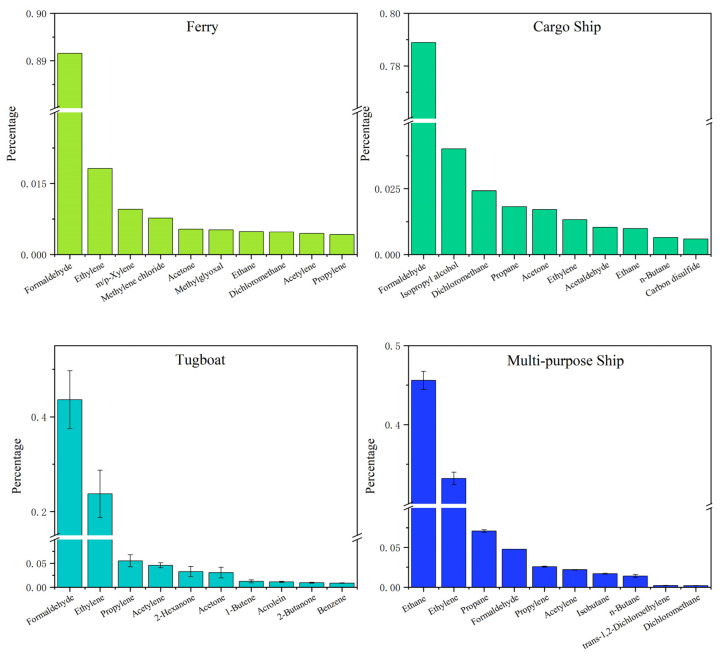
The top ten components with the highest percentages among the VOCs emitted by different types of ships.

Figure 3 lists the top ten VOC components in terms of each concentration percentage in total VOCs from the exhaust of four types of ships. The main VOCs emitted by the ferry included formaldehyde (89%), ethylene (1.8%), and *m/p*-xylene (1.0%); those from the cargo ship included formaldehyde (78.9%), isopropanol (4.0%), and dichloromethane (2.4%); those from tugboats included formaldehyde (43.6% ± 6.1%), ethylene (23.7% ± 5.0%), and propylene (5.5% ± 1.3%); and those from the multi-purpose ship included ethane (45.6% ± 1.1%), ethylene (33.2% ± 0.8%), and propane (7.1% ± 0.2%). Among the VOCs emitted by all ship types, formaldehyde contributed the most to OVOCs, while ethylene and propylene significantly contributed to alkenes. These results showed similar VOC compositions from cargo ships and speedboats to those reported in PRD studies [24]. Formaldehyde plays a critical role in atmospheric photochemistry and ozone formation and contributes to the photochemical cycling of atmospheric radicals (such as HO_x_·) and driving ozone formation [33,34,35]. Additionally, studies have shown that C_2_–C_4_ alkenes are the most important ozone precursors in VOCs in the PRD region [36].

**Figure 4:**
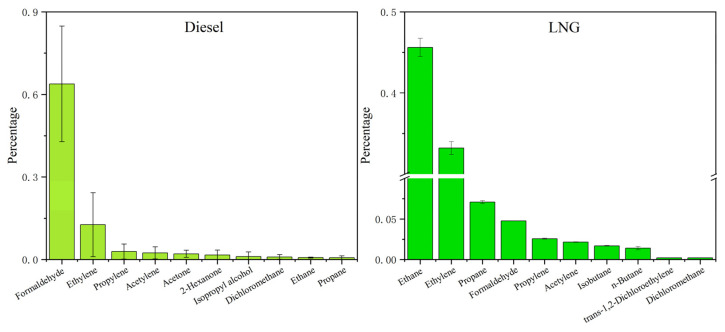
The top ten components of VOCs emitted by ships with different fuel types.

In this study, the ferry, cargo ship, and tugboats were fueled by diesel oil, while the multi-purpose ship used LNG (Table 1). For diesel-fueled ships, the dominant VOC components were OVOCs (71.9% ± 18.3%) (Figure 2), alkenes (16.9% ± 15.3%), and alkanes (4.4% ± 1.8%). By contrast, the top three components in VOCs emitted by the LNG-fueled ship were alkanes (56.3% ± 1.6%), alkenes (36.0% ± 0.9%), and OVOCs (5.0% ± 0.1%). Compared with diesel, the LNG-fueled ship showed significantly higher proportions of alkanes and alkenes, while the proportions of OVOCs and aromatic hydrocarbons decreased substantially. Figure 4 lists the top ten VOC components in terms of each concentration percentage in total VOCs from the exhaust of the two ship fuel types. Diesel-fueled ships mainly emitted formaldehyde (63.8% ± 21.0%), ethylene (12.7% ± 11.6%), and propylene (2.9% ± 2.7%), whereas the LNG-fueled ship primarily emitted ethane (45.6% ± 1.1%), ethylene (33.2% ± 0.8%), and propane (7.1% ± 0.2%). The characteristics of emitted VOCs from the LNG-fueled ship in this study were consistent with those of other LNG-powered vehicles [37], demonstrating dominant alkane species.

**Figure 5:**
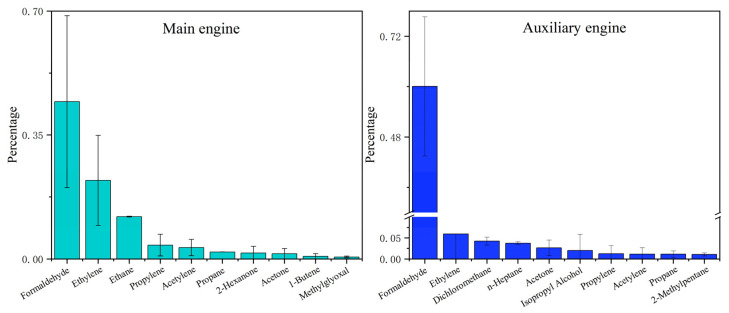
The top ten VOC components emitted by the main engines and auxiliary engines of ships.

The composition of VOCs emitted by ships was also closely related to engine power and combustion efficiency. OVOCs in diesel engines typically originate from the incomplete combustion processes of low molecular weight hydrocarbons; thus, engine type may significantly affect VOC emission characteristics [14,33]. Ship engine types include main engines (propulsion engines), auxiliary engines, and boilers [38]. In this study, the engine types for ship source sampling included the main engines and auxiliary engines. For the main engines, the top three components in emitted VOCs were OVOCs (50.0% ± 14.7%), alkenes (27.5% ± 11.3%), and alkanes (16.6% ± 1.3%). For auxiliary engines, the top three components were OVOCs (70.0% ± 12.2%), alkanes (12.0% ± 2.1%), and alkenes (8.3% ± 10.3%). Auxiliary engines had higher proportions of OVOCs than main engines, while main engines had higher proportions of alkenes and alkanes than auxiliary engines. The results of this study differed from those of Zhang et al. (2024), who reported alkanes as the most abundant VOCs in auxiliary engines [14]. This discrepancy was possibly due to a lack of measurements for OVOCs such as formaldehyde, acetaldehyde, and benzaldehyde in their study, leading to an underestimation of their contributions. Figure 5 lists the top ten VOC components in terms of each concentration percentage in total VOCs emitted by the two engine types, which both showed formaldehyde as the main species for main engines (44.4% ± 24.3%) and auxiliary engines (60% ± 16.6%).

### 3.2. Analysis of Ozone Formation Potential of Ship-Emitted VOCs

**Figure 6:**
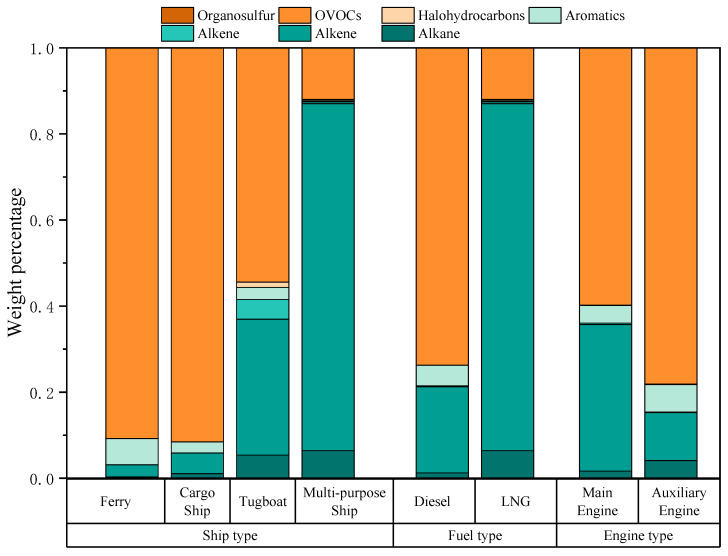
The ozone formation potential (OFP) of VOCs emitted by different types of ships. The OFP value of each species is presented as its percentage in the total VOC contributions.

The composition of VOCs emitted by ships varies significantly due to ship type, fuel used, and main/auxiliary engines, leading to different contributions of ship-source VOCs to O_3_ formation [14]. This study calculated the OFP of VOCs emitted by ships under various scenarios, presented in Figure 6. For the ferry, the main OFP contributors were OVOCs (90.8%), aromatic hydrocarbons (6.1%), and alkenes (2.8%); for the cargo ship, they were OVOCs (91.5%), alkenes (4.8%), and aromatic hydrocarbons (2.5%); for tugboats, the main OFP contributors were OVOCs (54.4% ± 7.4%), alkenes (31.6% ± 8.2%), and alkanes (5.4% ± 0.5%); and for the multi-purpose ship, the main OFP contributors were alkenes (80.6% ± 0.02%), OVOCs (12.0% ± 0.03%), and alkanes (6.4% ± 0.1%). As for different fuel types, diesel-fueled ships were dominated by OVOCs (73.7% ± 18.1%), alkenes (20.0% ± 17.2%), and aromatic hydrocarbons (4.8% ± 1.6%), while the LNG-fueled ship was dominated by alkenes (80.6% ± 0.02%), OVOCs (12.0% ± 0.03%), and alkanes (6.4% ± 0.1%). For engines, the main OFP contributors for main engines were OVOCs (59.8% ± 20.1%), alkenes (34.1% ± 19.4%), and aromatic hydrocarbons (4.1% ± 1.3%), and for auxiliary engines, they were OVOCs (78.1% ± 13.2%), alkenes (11.1% ± 12.4%), and aromatic hydrocarbons (6.4% ± 2.8%). In all, these results indicate that OVOCs were the primary OFP contributors for diesel-fueled ships, while alkenes dominated the OFP of the LNG-fueled ship. For different engine types, OVOCs and alkenes are the main OFP contributors for both main and auxiliary engines. In addition, the analysis of OFP contributions from individual species was consistent with the ranking results for VOCs, and the top ten VOC components all exhibited high OFP contributions. Species with major OFP contributions from OVOCs included formaldehyde, acrolein, and acetone; those from alkenes included ethylene and propylene; and those from aromatic hydrocarbons included *m/p*-xylene and 1,2,4-trimethylbenzene.

### 3.3. Contribution of Ship-Emitted VOCs to Ambient Atmospheric VOCs in Guangzhou

#### 3.3.1. Analysis of Air Mass Transport Trajectories at the Port Site

**Figure 7:**
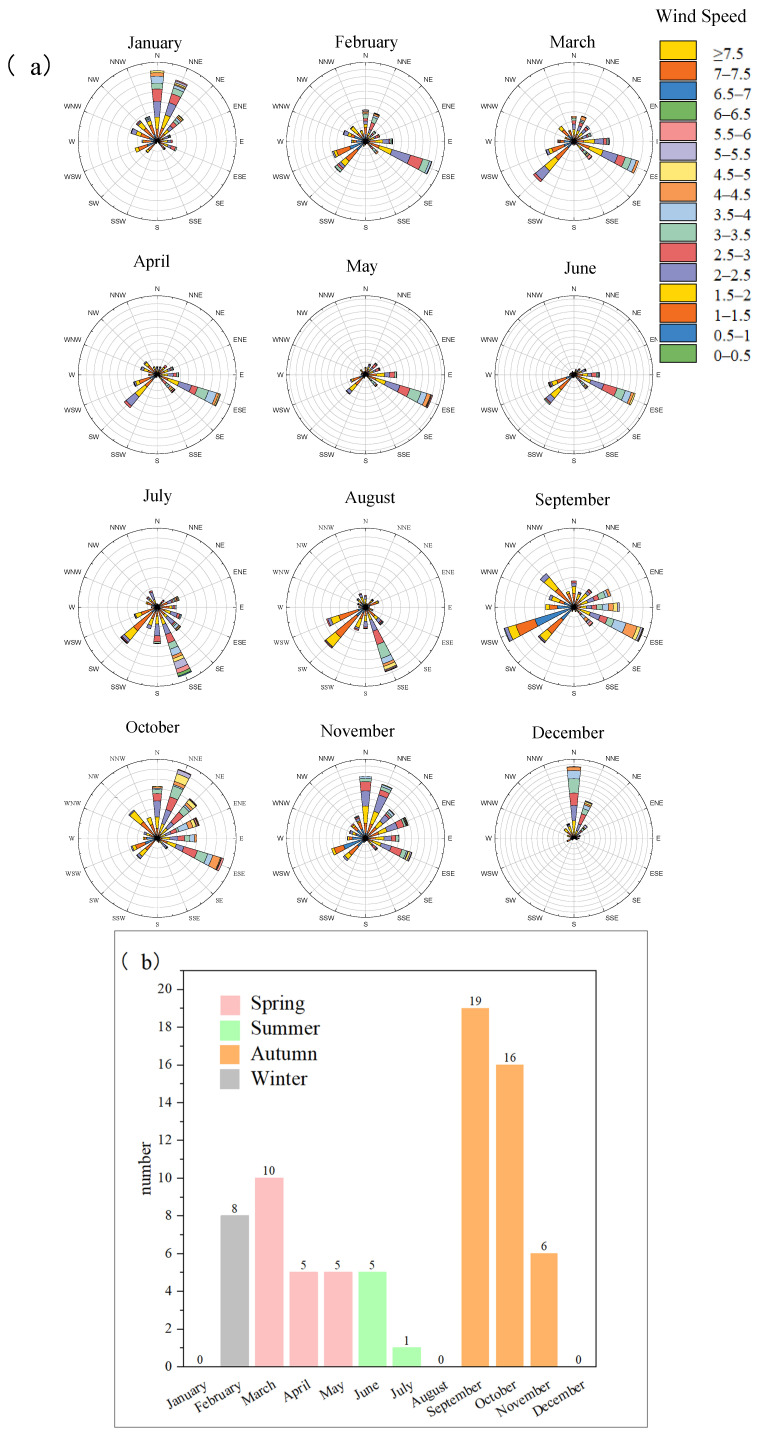
(**a**) The distribution of wind direction and wind speed in each month at the port site; (**b**) the number of ozone pollution days in each month at the port site.

To investigate the contribution of ship-emitted VOCs to ambient atmospheric VOCs in the port region, we performed field observations at a port site and an urban site. The real-time VOC concentrations at the port site were monitored for one year, while the same measurement was conducted at the urban site in July and September. The monthly characteristics of wind speed and wind direction during the port site sampling period are shown in Figure 7a. The number of ozone pollution days in each month at the port site is shown in Figure 7b. Ozone pollution days were identified through the National Ambient Air Quality Standards, defined as the maximum 8 h average ozone concentration exceeding 160 μg/m^3^ [39]. Ozone pollution days accounted for 20 days in spring, 6 days in summer, 41 days in autumn, and 8 days in winter. At the port site, southeast and south winds prevailed from February to September, while north winds dominated from October to January. Considering that the port is located south of Guangzhou, and south winds can transport marine air masses to the port and inland areas, the impact of ship emissions on ambient VOCs in both the port and urban areas is expected to be most significant between February and September. However, the occurrences of ozone pollution in Guangzhou from April to August were very low (Figure 7b), suggesting that, even under the influence of ship-emitted VOCs in the port and urban areas, the formation of O_3_ pollution is still determined by multiple factors.

**Figure 8:**
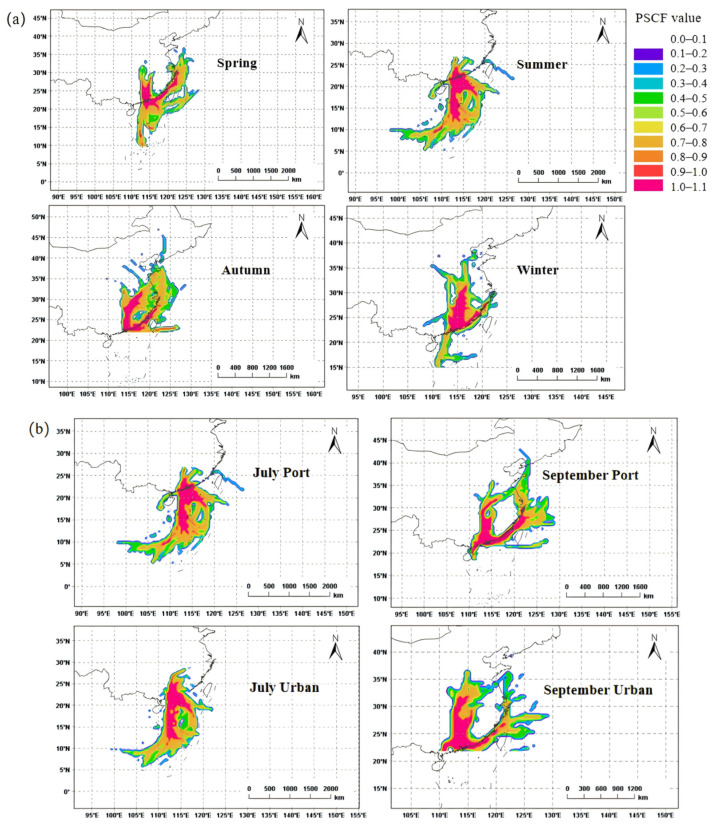
(**a**) PSCF analysis associated with VOC concentrations in four seasons at the port site; (**b**) PSCF analysis associated with VOC concentrations in July and September at the port site and urban site.

In this study, the PSCF analysis was conducted using backward trajectories to further elucidate the influence of ship plumes on the port and urban sites (as shown in Figure 8a). The potential source of air masses arriving at the port site was discussed according to four seasons: spring (March–May), summer (June–August), autumn (September–November), and winter (December–February) [40,41]. During spring, air masses at the port site mainly originated from inland regions in the north, with a small portion transported along the coastline from the northeast to the port area. Northeast air masses along the coastline may also carry ship-emitted VOCs from Fujian ports to Guangzhou Port, while northern air masses primarily transport anthropogenic VOCs to urban Guangzhou and the port region. In summer, all air masses at the port site originated from the southern marine area, where both marine air masses and exhaust emissions from port-docked ships significantly influenced the port and urban sites. The air mass sources in autumn and winter were similar to those in spring, with VOC emissions closely linked to air masses from the north and northeast of Guangzhou, leading to a lower contribution of ship exhaust in autumn and winter than in summer.

Additionally, the potential sources of VOCs at the urban and port sites in July and September were both analyzed to illustrate air mass source characteristics (Figure 8b). In July, the sites were both completely influenced by marine air masses, while, in September, they were affected by both coastal air masses (46.2%) and inland air masses (53.8%). The discrepancy between the contribution of marine-transported air masses and the occurrence of ozone pollution (Figure 7b) suggested that the association between increased VOC contributions from ship emissions and ozone pollution was not a simple linear response.

#### 3.3.2. Source Apportionment of Ambient Atmospheric VOCs and the Contribution of Ship-Emitted VOCs

**Figure 9:**
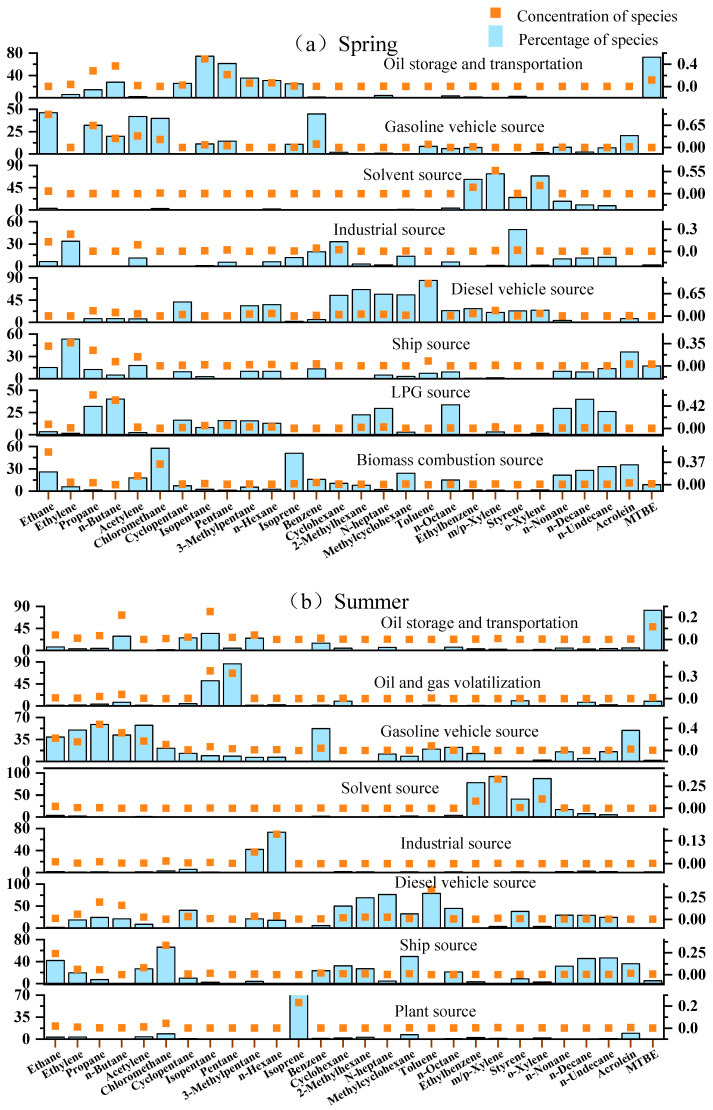

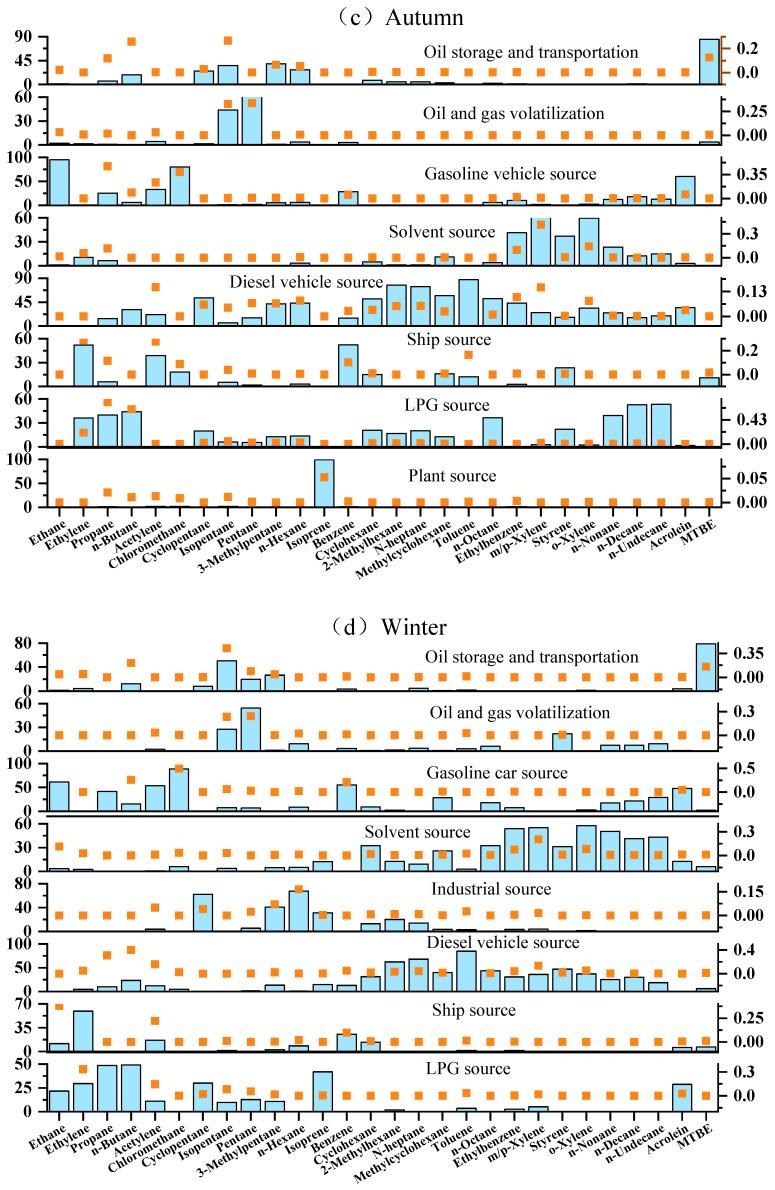
Source apportionment of ambient atmospheric VOCs in the four seasons at the port site. The orange dots represent the concentrations of species (right y-axis, ppb), and the blue bars represent the percentage of species (left y-axis, %).

The emission characteristics of VOCs from diesel-fueled ships were chosen as the identification criteria to resolve the contributions of ship-emitted VOCs via PMF analysis (Figure 9). The source apportionment at the port site was conducted according to four seasons: spring (March–May), summer (June–August), autumn (September–November), and winter (December–February) [40,41]. The source apportionment classified VOCs from 10 sources, including gasoline vehicle sources, diesel vehicle sources, ship sources, solvent sources, industrial sources, biogenic sources, biomass burning sources, liquefied petroleum gas (LPG) sources, oil and gas volatilization sources, and oil storage and transportation sources.

The characteristic VOC species used to identify each source are listed in Figure 9. Generally, gasoline vehicle sources were dominated by ethane, propane, acetylene, benzene, chloromethane, and acrolein. Diesel vehicle sources were dominated by hexane, n-heptane, n-octane, toluene, and other long-chain alkanes and aromatic hydrocarbons [15,42]. Solvent sources were dominated by aromatic hydrocarbons such as toluene, *m/p*-xylene, and *o*-xylene [43,44]. Industrial sources were dominated by styrene, 3-methylpentane, n-hexane, and other long-chain alkanes and aromatic hydrocarbons [44,45]. Biogenic sources were dominated by isoprene (Liu et al., 2021) [43]. Biomass-burning sources were dominated by ethane, chloromethane, and long-chain alkanes [46]. LPG sources were dominated by propane and butane [47]. Oil and gas volatilization sources were dominated by propane, pentane, and butane [47,48,49,50]. Oil storage and transportation sources were dominated by isopentane and methyl tert-butyl ether (MTBE) [51]. Ship sources were dominated by ethylene, acetylene, acrolein, and long-chain n-alkanes, including n-octane, n-nonane, n-decane, and n-undecane. Notably, although formaldehyde is the major emitted OVOC from ship exhaust, the most commonly used instrument for the online measurements of VOCs could not detect formaldehyde. Thus, the PMF analysis did not include formaldehyde to determine the ship emission source.

**Figure 10:**
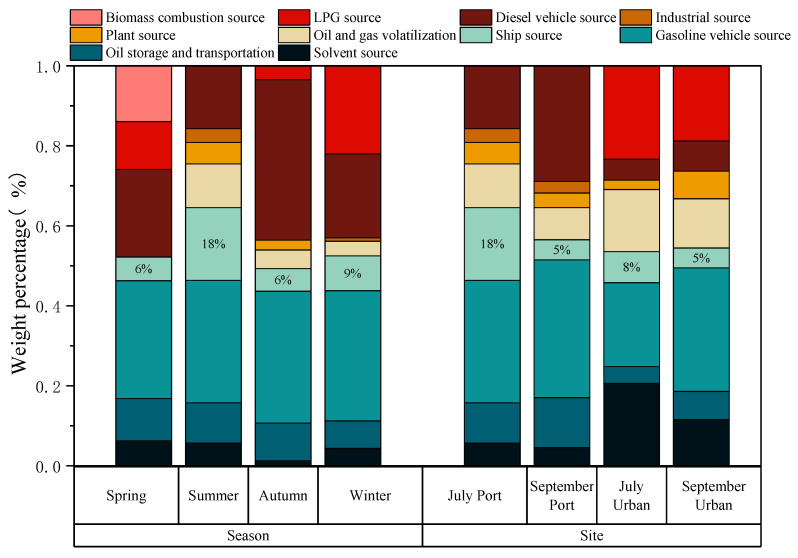
The contributions of different sources to ambient VOCs at the port and urban sites.

The contributions of different sources to ambient VOCs at the port and urban sites are shown in Figure 10. In spring, the main contributors at the port site were gasoline vehicle sources (29.5% ± 4.6%) and diesel vehicle sources (21.9% ± 4.1%); in summer, gasoline vehicle sources (30.6% ± 1.7%) and ship sources (18.2% ± 0.8%) dominated the VOC emission sources; in autumn, ambient VOCs were mainly from diesel vehicle sources (40.1% ± 5.1%) and gasoline vehicle sources (33.0% ± 4.7%); in winter, the main sources of VOCs were gasoline vehicles (32.6% ± 7.4%), diesel vehicles (21.0% ± 6.2%), and LPG (22.0% ± 3.5%), which were associated with traffic emissions. Given the seasonal sources of VOCs, ship sources showed the highest contribution in summer (18.2% ± 0.8%), consistent with the PSCF analysis, indicating that the port site was primarily influenced by marine air masses in summer. Ship sources in the other three seasons were as follows: winter, 8.7% ± 1.9%; spring, 6.0% ± 1.1%; and autumn, 5.6% ± 1.7%. The contribution of ship sources in this study was similar to those reported in studies conducted from January to December 2021 in the PRD [15].

The VOC emission sources at the port and urban sites differed in July and September (Figure 10). In July, the main VOC sources at the port site were gasoline vehicles (30.6% ± 1.7%) and ships (18.2% ± 0.8%), while the urban site was dominated by LPG (23.4% ± 4.3%), gasoline vehicles (20.96% ± 4.2%), and solvents (20.60% ± 2.5%). In September, the port site was primarily influenced by gasoline vehicles (34.5% ± 3.9%) and diesel vehicles (28.9% ± 4.2%), whereas the urban site was dominated by gasoline vehicles (30.9% ± 8.3%) and LPG (18.8% ± 5.1%). Ship source contributions were 18.2% ± 0.8% at the port site but decreased to 7.8% ± 1.5% at the urban site in July, and both sites showed a 5.0% contribution of ship sources to ambient VOCs in September. The higher contribution of ship sources to ambient VOCs at the port site in July suggests that, when south winds prevail, coastal areas near the port are more easily influenced by ship sources, and the contribution of ship-related emission air masses to atmospheric VOCs decreases as they move inland.

Overall, this study found that OVOCs dominated the VOCs emitted by diesel-fueled ships, while VOCs from an LNG-fueled ship primarily comprised alkanes. Given the active role of OVOCs in atmospheric photochemical reactions, ship-emitted VOCs may be a significant influence on O_3_ formation, especially during summer, when south winds prevail, increasing the contribution of ship exhaust VOCs and the potential occurrence of O_3_ pollution. Therefore, the impact of ship emission sources should be considered when formulating VOC control strategies in port cities. Additionally, due to the difficulty of directly sampling ship exhaust and the diversity of ship types, the representativeness of the reported ship-source VOC characteristics in this study may be challenged, mainly reflecting the emission characteristics of small-to-medium diesel-fueled ships in the port area. Further sampling and analysis of VOCs emitted by large container ships and heavy-oil-fueled ships will be conducted.

## 4. Conclusions

This study characterized the compositions of VOCs obtained from four types of ships in the Guangzhou Port area, analyzing the localized source profile characteristics of ship-emitted VOCs with respect to different vessel uses, fuel types, and engine operating conditions. The dominant VOC components in ferry emissions were OVOCs (91.1%), which also dominated the VOCs in exhaust from a cargo ship (OVOCs, 87.0%) and tugboats (OVOCs, 54.4% ± 7.9%); conversely, alkanes were the major VOCs from a multi-purpose ship (56.3% ± 1.6%). The ferry, cargo ship, and tugboats were all fueled by diesel, while the multi-purpose ship was fueled by LNG, suggesting that OVOCs account for more than 50% of VOCs from diesel-type ships. Furthermore, formaldehyde was the most abundant OVOC, and ethylene and propylene were the primary contributors to alkenes. In addition, OVOCs were still the dominant VOC component under the main and auxiliary engine operation conditions. The OFP estimation showed that diesel-fueled ships were dominated by OVOCs (73.7% ± 18.1%), while the LNG-fueled ship was dominated by alkenes (80.6% ± 0.02%), indicating that OVOCs are the primary OFP contributors for diesel ships, and alkenes dominate the OFP of LNG ships. The field observations of ambient VOCs were conducted at both the port and urban sites, and the PMF source apportionment showed that ship-source contributions at the port site were highest in summer (18.2% ± 0.8%), consistent with PSCF analysis suggesting the driving factor of marine air mass in summer. The comparative analysis between the port and urban sites showed that the ship source contribution was higher at the port site (18.2% ± 0.8%) than at the urban site (7.8% ± 1.5%) in July, but both sites showed similar contributions (5.0%) in September. These results suggest that coastal areas near the sea are more frequently influenced by ship exhaust during summer when south winds prevail, with reduced contributions as ship-related air masses move inland. In summary, this study provides empirical evidence regarding ship-emitted VOC contributions to atmospheric VOCs in port cities and their potential impact on ozone formation. These findings offer an approach to comprehensively assess the impacts of ship-source VOCs in port cities and formulate effective control strategies.

## Figures and Tables

**Table 1:** Information for four types of ships in Guangzhou Port.

Ship Type	Fuel Type	Tonnage, t
Ferry	Diesel	750
Cargo ship	Diesel	2156
Tugboat 1	Diesel	351
Tugboat 2	Diesel	323
Multi-purpose ship	LNG	2388

## Data Availability

The observational data obtained in this study, including the VOC data and meteorological parameters, are available from the corresponding authors upon reasonable request (chengcl.vip@foxmail.com).
